# Consumption‐based Material Flow Accounting: Austrian Trade and Consumption in Raw Material Equivalents 1995–2007

**DOI:** 10.1111/jiec.12055

**Published:** 2013-09-30

**Authors:** Anke Schaffartzik, Nina Eisenmenger, Fridolin Krausmann, Helga Weisz

**Affiliations:** Institute of Social Ecology, Schottenfeldgasse 29, A-1070, Vienna, Austria

**Keywords:** environmental input‐output analysis, globalization, industrial ecology, material flow accounting (MFA), raw material equivalents (RME), resource use indicator

## Abstract

In 2007, imports accounted for approximately 34% of the material input (domestic extraction and imports) into the Austrian economy and almost 60% of the GDP stemmed from exports. Upstream material inputs into the production of traded goods, however, are not yet included in the standard framework of material flow accounting (MFA). We have reviewed different approaches accounting for these upstream material inputs, or raw material equivalents (RME), positioning them in a wider debate about consumption‐based perspectives in environmental accounting. For the period 1995–2007, we calculated annual RME of Austria's trade and consumption applying a hybrid approach. For exports and competitive imports, we used an environmentally extended input‐output model of the Austrian economy, based on annual supply and use tables and MFA data. For noncompetitive imports, coefficients for upstream material inputs were extracted from life cycle inventories. The RME of Austria's imports and exports were approximately three times larger than the trade flows themselves. In 2007, Austria's raw material consumption was 30 million tonnes or 15% higher than its domestic material consumption. We discuss the material composition of these flows and their temporal dynamics. Our results demonstrate the need for a consumption‐based perspective in MFA to provide robust indicators for dematerialization and resource efficiency analysis of open economies.

**Supplementary Information:**

The online version of this article (doi:10.1111/jiec.12055) contains supplementary material, which is available to authorized users.

## Introduction

Globalization has resulted in increasing import and export volumes, in structural changes within the trading economies, and in an increasing spatial differentiation of global production and consumption activities (WTO 2008; Peters et al. 2011). Nonetheless, most standardized national energy, material, or emission inventories continue to take on a domestic or production‐based perspective. Under the existing framework, resources used and emissions generated in the production of imported goods are attributed to the exporting country and not to the country of final consumption. For open economies, such production‐based inventories increasingly fail to adequately quantify the life‐cycle–wide environmental pressures associated with domestic consumption. In recognition of the increasing importance of international trade for environmental accounting and policy, complementary approaches have been developed and range from the energy costs approach of the 1970s (Bullard and Herendeen 1975) to the more recent consumption or footprint approaches and energy and greenhouse gas emissions accounting (Munksgaard and Pedersen 2001; Peters 2008; Hertwich and Peters 2009). Analogous considerations apply to economy‐wide material flow accounts and derived national material flow accounting (MFA) indicators (Eurostat 2001). In the course of the production processes of material goods, a high amount of raw materials is consumed, transformed into wastes and emissions, and not physically included in the final product itself (Fischer‐Kowalski and Amann 2001).

**Figure 1:**
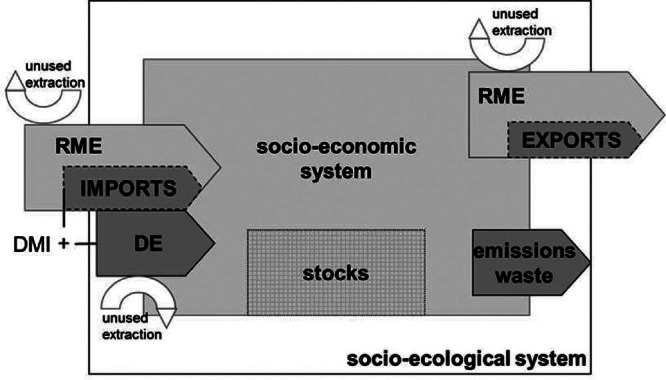
The role of raw material equivalents (RME) in the material flow accounting framework. Direct material input (DMI) is obtained by adding imports and domestic extraction (DE) and subtracting exports.

Although, from a consumption perspective, the materials used in the production process of an exported good should be allocated to the importing economy, standard MFA indicators, such as direct material input (DMI) or domestic material consumption (DMC), follow a production‐based approach. In assessing resource efficiency and dematerialization, the usefulness of these indicators is limited by the fact that a reduction in the indicator value can be either the result of real improvements or to a relocation of material‐intensive production stages to other countries. Attempts to develop consumption‐based approaches for MFA date back to the 1990s. Within the Eurostat framework, the concept of raw material equivalents (RME) was proposed to allow for the inclusion of upstream material inputs into the production of imports and exports (Eurostat 2001) (figure 1).

Although the development of methods to account for RME could profit from the methodological achievements in the fields of energy and carbon footprints, empirical applications of the RME concept and comprehensive RME accounts are still rare. This is largely a result of the complex and, at the same time, insufficiently documented nature of socioeconomic material use. However, in the past few years, several empirical RME studies have been published using different calculation methods (see below). In this article, we present the first calculation of annual RME of trade and material consumption for Austria from 1995 to 2007. We used a hybrid approach combining an environmentally extended input‐output (IO) model with coefficients from life cycle inventory (LCI).

**Figure 2:**
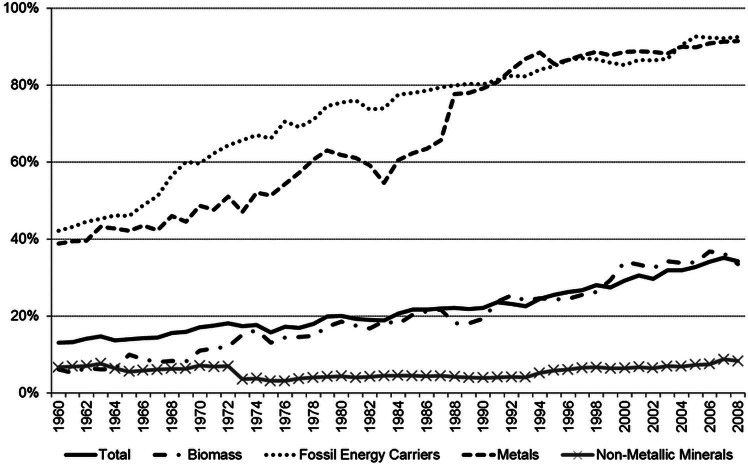
Share of imports in Austria's direct material input (DMI), 1960–2008. Source of data: Statistik Austria (2011b).

Austria serves as a typical example for the growing significance of trade flows in the metabolism of industrial economies: From 1960 to 2008, the share of imports in direct material input grew from 16% to 34%; only two thirds of all material input was of domestic origin. Twenty‐three percent of all materials processed in Austria leave the country as exports (Statistik Austria 2011b; Eisenmenger et al. 2011). Biomass and nonmetallic minerals in particular are still mainly extracted domestically. Fossil fuels (FFs) and many important metals are not (or no longer) available within Austria, so that domestic extraction has either ceased or is decreasing. However, the strategic importance of these minerals leads to continuous demand and a growing dependence on imports (figure 2).

This article is composed of two parts: The first provides an overview of the different methods currently used in RME calculation as well as a detailed description of our hybrid approach. The second part is devoted to the presentation of the results obtained for the RME of Austrian trade and consumption.

## Methods of Raw Material Equivalents Calculation

The different approaches to RME calculation currently being developed and used can be classified into (1) IO approaches, (2) coefficient or LCI‐based approaches, and (3) hybrid IO approaches.

### Input‐Output Approaches

IO approaches use standardized monetary (and very seldom physical) IO tables compiled as part of national accounts. The advantages of using IO data to quantify RME are (1) systemic coverage of the whole economy, (2) high compatibility with economy‐wide MFA data as well as the United Nations’ (UN's) System of Environmental‐Economic Accounting, and (3) integration into the standard system of national accounting. Interindustry flows and final demand are usually reported in monetary, rather than in physical, terms. For the often highly aggregated activities, this can lead to distorted allocation of physical flows. For example, in the Austrian IO tables, livestock as well as crop production are included as part of the same agricultural activity. However, both require quantitatively and qualitatively very different physical inputs. The IO approach has been used in two distinct ways to calculate RME.

A single‐region IO (SRIO) model applies only one IO table (that of the importing economy) using information on domestic interindustry relations and technical coefficients to calculate the RME of exports as well as of imports. The underlying assumption is that domestic quantity and mix of inputs can also be applied to the economies from which the product is imported. The resulting RME of imports approximate the change in demand that would arise if the country were to produce the imported goods domestically. They thus provide an indicator of virtual resource savings or losses. This approach was applied in the first RME accounts (e.g., Weisz et al. 2008; Weisz 2006) and then again more recently in a study of a selection of Latin‐American countries by Muñoz and colleagues (2009). Especially when it is applied to small, open economies, where certain goods are not produced (or some raw materials are not available for extraction) and relevant production processes are not or only poorly reflected in the national IO data, the results obtained differ greatly from the actual material inputs required for the production of traded goods.

In multiregional IO (MRIO) models, this shortcoming of the SRIO model is solved by integrating IO data from other countries or regions into one IO table for the whole world.^1^ This approach can be considered as the most advanced in terms of depicting the specific (monetary) interindustry relations in the economies producing the imported goods. At the same time, the differences in the number of activities reported require harmonization. Because of the large amount of data involved, the calculation of RME with MRIO usually requires major processing power and long‐term dedication. Comprehensive MRIO models need to contain IO data for a large number of countries. The complexity is sometimes reduced by either combining country‐specific IO data for the most relevant trade partners and with an average IO table for a “rest of the world” region or by using average regional IO tables. An MRIO calculation of RME using the Global Resource Accounting Model was presented by Bruckner and colleagues (2012) and by Wiebe and colleagues (2012) with a focus on emerging economies. Other examples of MRIO models include those based on the Global Trade Analysis Project (Narayanan et al. 2012), the multiregional environmentally extended supply and use/input‐output EXIOBASE (Tukker et al. 2009) and its follow‐up CREEA, the multiregion IO database Eora (Lenzen et al. 2012), and the World Input‐Output Database (Timmer 2012). In contrast to the SRIO approach, MRIO‐derived RME strive to represent the actual material inputs associated with the production of traded goods.

### Coefficient or Life Cycle Inventory–Based Approaches

Coefficient‐based approaches use product‐ or resource‐specific coefficients to assess upstream material inputs. Whereas the IO‐based approaches seek to reach economy‐wide coverage, the coefficient approaches tend to focus on certain products or product groups in a bottom‐up manner. Coefficients used are usually derived from LCIs. These inventories cover a large number of products and processes in great detail and, in contrast to IO data, are built from information on physical (and not monetary) flows. The main challenge here lies in ensuring full coverage while avoiding double counting.^2^ The coefficient approach was developed and applied by the Wuppertal Institute based on material intensity analysis by Ritthoff and colleagues (2002). The aggregate indicator derived from these accounts is the total material requirement (TMR), which—unlike the other approaches to RME calculation presented here—also includes unused extraction.^3^ TMR accounts allocate upstream requirements to material groups based on what the inputs are required for: All materials used for producing biomass products are classified as “biomass” no matter whether they are fossil energy carriers or nonmetallic minerals used in infrastructure for biomass production. Thus, results cannot be directly compared. For an application of the TMR approach, please refer to the work by Dittrich and colleagues (2012).

### Hybrid Approaches

The term “hybrid approach” is used here to refer to the combination of IO and LCI data as developed by the LCI community to solve system boundary issues (Suh et al. 2003). In RME applications, an SRIO approach is extended by an LCI module for noncompetitive imports, (i.e., for those products or activities that are not, or not sufficiently, represented by the domestic IO structures). The RME calculation for Austria presented in this article is based on a hybrid approach and described in detail below. The Austrian approach is highly comparable to calculations performed for Germany (Buyny et al. 2009) and the Czech Republic (Weinzettel and Kovanda 2009) and, most recently, for the EU27 (Schoer et al. 2012).

## A Hybrid Method for Calculating Raw Material Equivalents

In calculating the RME of Austria's trade from 1995 to 2007, the RME of exports and competitive imports^4^ were computed using an environmentally extended open static IO model, whereas for noncompetitive imports, we extended this model using coefficients from LCI.

### The Input‐Output Module: Raw Material Equivalents of Exports and Competitive Imports

The model we used is based on a generalized form of the open static IO model (Leontief 1941, 1986; Lenzen 2001; Miller and Blair 2009), for which we will briefly restate the basic equations. We followed the industry technology assumption presuming each industry to employ a certain technology for all goods it produces. The matrix of intermediate input coefficients **B** (product × industry) was calculated as the share of the intermediate inputs in the industry output, whereas the matrix of market shares **D** (industry × product) depicts the share of each industry in the total domestic production of each product. The multiplication **B** with **D** yields the n × n (technology) matrix of interindustry requirements **A** (product × product). Let **x** be the n × 1 vector of total output of the economy, **A** the n × n (technology) matrix of interindustry requirements, and **y** the n × 1 vector of final demand. Equation 1 states, in standard matrix notation, that the total output of the economy is the sum of all intermediate and final consumption.

1 $$ \mathbf{x}=\mathbf{Ax}+\mathbf{y} $$

Solving for total output x yields equation 2:

2 $$ \mathbf{x}={\left(\mathbf{I}-\mathbf{A}\right)}^{-1}\mathbf{y} $$

where **I** is the n × n identity matrix and (**I** − **A**)^−1^ is the n × n matrix of total requirements or the Leontief inverse. Each element of the Leontief inverse {l_ij_} shows the total (i.e., the direct and indirect) intermediate requirements of activity i in producing one unit of final demand of activity j.

This model can be extended to include any kind of factor inputs required to produce a given final demand (Lenzen 2001). For the purpose of this article, we consider *material* factor inputs. Let **F** be the k × n matrix of material‐use intensities where k is the number of materials considered. Each element {f_αi_} shows the *direct* input of material α into the activity i per unit of output of that activity. The *total* (i.e., direct and indirect) material requirements **E** (k × n matrix with elements {e_αi_}) embodied in final demand is given by equation 3:

3 $$ \mathbf{E}=\mathbf{F}{\left(\mathbf{I}-\mathbf{A}\right)}^{-1}\mathbf{y} $$

The term F (I − A)^−1^ is also called the multiplier (see Lenzen 2001 for an analysis of different multipliers). Because we are interested in trade flows, we decompose final demand **y** into the two components, foreign final demand (**y**^**ex**^) and domestic final demand (**y**^**d**^), and calculate the direct and indirect material factor inputs of k materials needed to produce a given vector of exports (**y**^**ex**^) as shown in equation 4.

4 $$ {\mathbf{E}}^{\mathbf{ex}}=\mathbf{F}{\left(\mathbf{I}-\mathbf{A}\right)}^{-1}{\mathbf{y}}^{\mathbf{ex}} $$

The direct and indirect material factor inputs for competitive imports are calculated using the same multiplier. By adding the direct and indirect material requirements of exports and competitive imports to the mass of the traded goods themselves, the RME of exports and competitive imports are calculated.

### The Life Cycle Inventory Module: Raw Material Equivalents of Noncompetitive Imports

Based on production statistics, we identified the following commodity groups as major noncompetitive imports for the Austrian economy: products from the extraction and first processing of metals (iron, copper, and aluminium); the processing of raw materials for fertilizer production; and petroleum and gas extraction. For these imports, the national IO statistics lack the technology information and commodity resolution for the calculation of RME (see “Input‐Output Approaches” above and the Supporting Information available on the Journal's Web site). The LCI module consists of coefficients in tonnes/tonne extracted from GEMIS based on the principle origin of Austrian imports (Öko‐Institut 2009), which express the upstream inputs of noncompetitive imports. LCI coefficients have a higher resolution of commodity groups than the IO tables: For example, iron, copper, and aluminium are aggregated to one commodity group in the IO table, whereas the LCI provides much more disaggregated coefficients for each of these metals. In order to bridge the different levels of aggregation, we disaggregated the respective import flows in the IO module using United Nations (UN) Comtrade data at the three‐digit level of the Standard International Trade Classification (SITC; Rev.3), which distinguishes 261 different products.^5^ For example, in 2000, 61% of Austria's iron imports (mass) were iron ore and concentrates, whereas 39% were products of iron and steel. The LCI coefficients applied to iron were selected from the LCI (Öko‐Institut 2009) and then weighted according to the composition of Austria's iron imports (i.e., 0.61*coefficient for iron ore and 0.39*coefficient for iron and steel products). The calculated upstream material inputs were then aggregated to the four main MFA categories.

**Figure 3:**
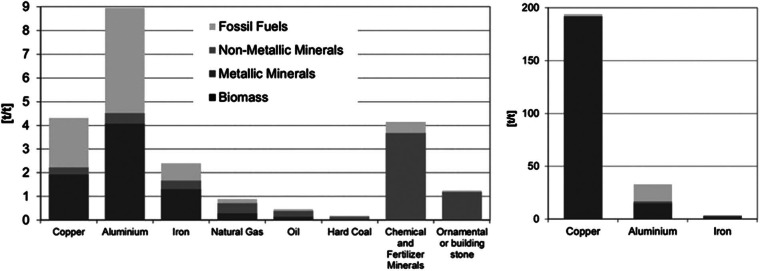
Selection of weighted input coefficients by material flow accounting categories (left, excluding waste rock) and inputs coefficients of metal production in gross ore (right, including waste rock) in tonnes per tonne (t/t). Source of data: Öko‐Institut (2009).

Figure 3 shows the calculated weighted and aggregated coefficients for a selection of noncompetitive imported goods. Following the Eurostat MFA methodology, extraction of metals is calculated in gross ore, that is, including waste rock (Eurostat 2001, 2012).^6^ A low average ore grade of approximately 1% translates, as visible in figure 3 for the case of copper, into comparably higher coefficients for upstream material inputs and a dominance of waste rock in their composition. We used average ore grades published by the U.S. Geological Survey (copper 1%, iron 50%, and aluminium/bauxite 25%). In order to demonstrate the varying degree of dominance of waste rock in the upstream material inputs, waste rock is excluded on the left‐hand side of figure 3.

Because the RME of noncompetitive imports are indirectly used in the Austrian economy in the production for final demand, we finally reintroduced the flows as calculated in the LCI module into the IO module.

### Data and Data Preparation

Supply and use tables (SUTs) are published annually for the Austrian economy by Statistics Austria (Statistik Austria 2011a). We used version A, which includes imports in the use and final demand tables. All entries are in monetary units and current prices. The SUTs for Austria are symmetric with a resolution of 57 activities/commodities. We calculated the direct input coefficient matrix **A** in the dimension commodity × commodity by applying equation 5, where **B** is a 57 × 57 matrix containing the inputs of commodity i per unit of output from industry j in the dimension commodities × industries, and **D** is the 57 × 57 matrix of market shares, in the dimension industries × commodities, containing for each commodity j the market share of each industry i (see ÖSTAT 1994; Kolleritsch 2004; Miller and Blair 2009).

5 $$ \mathbf{A}=\mathbf{BD} $$

The data input for matrix **F** (see above), expressing the material‐use intensities of each activity, is a specific disaggregation of DMI including the results of the LCI module measured in tonnes and total output **x** measured in Euros (Buyny et al. 2009; Wiedmann et al. 2006). Thus, all entries in matrix **F** are in the unit [t/€].

DMI is an indicator derived from economy‐wide material flow accounts (Eurostat 2012). DMI is composed of domestically extracted raw materials and total imports, all measured in tonnes per year. Data on the domestic extraction (DE) of raw materials are provided in a disaggregation of 65 material groups (Statistik Austria 2011b), according to the latest version of MFA standard tables from Eurostat (Eurostat 2012). A breakdown of material flows by activities (as in the National Accounting Matrix with Environmental Accounts) is not provided by statistical offices. We therefore carried out an allocation of DMI. For the DE component of DMI, we considered the producing primary activity to correspond to the activity using the raw material as factor input. This allocation to agriculture, forestry, and mining is a straightforward procedure, with the exception of construction minerals. In Austria, approximately half of the nonmetallic minerals used for construction purposes are extracted directly by construction and not the mining activity (Milota et al. 2011). We therefore allocated 50% of construction minerals DE to the mining and 50% to construction activity.

The import component of DMI was allocated to the economic activities with the help of annual matrices calculated on the basis of disaggregated UN Comtrade data. This step was necessary because products and activities in the Austrian IO table follow the CPA/NACE (Classification of Products by Activity/Nomenclature statistique des activités économiques dans la Communauté européenne) classification system (see the Supporting Information on the Web), for which no unambiguous correspondence to the MFA material groups exists. We extracted UN Comtrade data (SITC Rev.3) in physical units for Austrian imports between 1995 and 2007 at the most detailed level (AG5) for unambiguous allocation to NACE sectors of activity. Using this data, we calculated the share of an import flow (by MFA category), which had to be allocated to each economic activity.^7^

### Indicators

Using the hybrid method as described, we calculated the RME of imports (RIM = **F** (**I** − **A**)^−1^ imp) and of exports (REX = **F** (**I** − **A**)^−1^**y**^**ex**^). The RIM and REX include the trade flows themselves as well as upstream inputs into their production (cf. Eurostat 2001). Based on RIM, REX, and DE as available from Austrian MFA, we also calculated the traditional MFA indicators in their RME: raw material trade balance (RTB); raw material consumption (RMC); and raw material input (RMI; see table 1). The calculation presented here is analogous to the standard calculation of MFA indicators. Because our calculation of the RME is based on **E** = **F** (**I** − **A**)^−1^**y** (see equation 3), it is also possible to calculate RMI by substituting total final demand for **y** and to calculate RMC by substituting domestic final demand for **y**.

**Table 1:** Indicators derived from economy‐wide accounts of material flows and of raw material equivalents

*MFA indicators*	*RME‐based indicators*
Direct material input (DMI)	Raw material input (RMI)
= domestic extraction (DE) + imports	= DE + RME of imports (RIM)
Physical trade balance (PTB)	Raw material trade balance (RTB)
= imports – exports	= RIM – RME of exports (REX)
Domestic material consumption (DMC)	Raw material consumption (RMC)
= DE + imports – exports	= DE + RIM – REX

*Note:* MFA = material flow accounting; RME = raw material equivalents.

The upstream inputs into the production of traded goods maintain their material category and are not allocated to the material category to which the traded good belongs (also see above).

## Raw Material Equivalents of Austrian Trade: Empirical Results

Using the hybrid method, we calculated the RME of Austria's traded goods for the years 1995–2007 by material categories: biomass; metal ores; nonmetallic minerals; fossil energy carriers; and other products.^8^

### Austria's Imports and Exports

**Figure 4:**
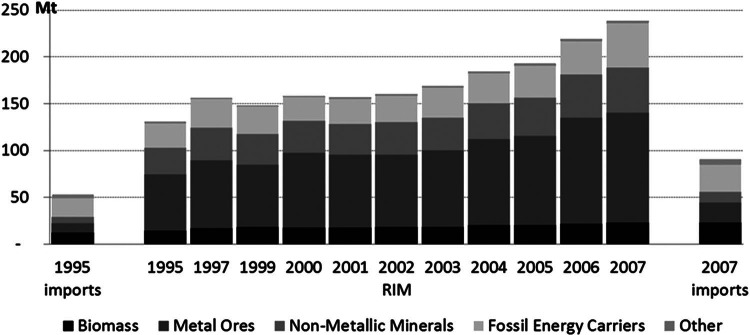
Austria's imports and RME of imports (RIM) in million tonnes (Mt).

In 2007, Austria imported approximately 91 million tonnes of material.^9^ The material inputs associated with the production of these imports in the economies of origin amounted to 239 million tonnes. RME of imports (RIM) hence surpassed imports by a factor of 2.6 (see figure 4). Whereas this ratio was almost identical in 1995 (factor of 2.5), the total volume of imports only amounted to 58% of the 2007 value. The largest fractions in Austria's 2007 imports were fossil energy carriers (31%), biomass (25%), and metal ores (23%).

In RIM, metal ores made up the largest share (49%), followed by fossil energy carriers (20%) and nonmetallic minerals (20%). RIM of metals were 5.5 times higher than metal imports in 2007. Metal ores are mainly traded as concentrates or products. From extraction^10^ to concentration and processing in final products, a steady reduction of the material mass occurs. The incorporation of large amounts of metal (especially steel) in production technologies and infrastructures further contributes to the disproportional significance of metal ores in RIM. Fossil energy carriers are important (energy) inputs in all other production processes. Therefore, their RIM are also significantly higher (factor of 1.7) than the imports. Austria's nonmetallic mineral RIM were 4.1 times larger than the imports because they are required in large amounts in production and transportation infrastructure. Between 1995 and 2007, relative growth of Austria's RIM (factor of 1.8) and imports (factor of 1.7) was almost identical.^11^ This was the aggregate effect of changes in the composition of imports and in the upstream material inputs as generated by our IO model.

In 2007, Austria exported approximately 58 million tonnes and, untypically of an industrialized economy, biomass made up the largest share of these exports (38%). Highly processed metal goods are the second‐largest export group (24%).

**Figure 5:**
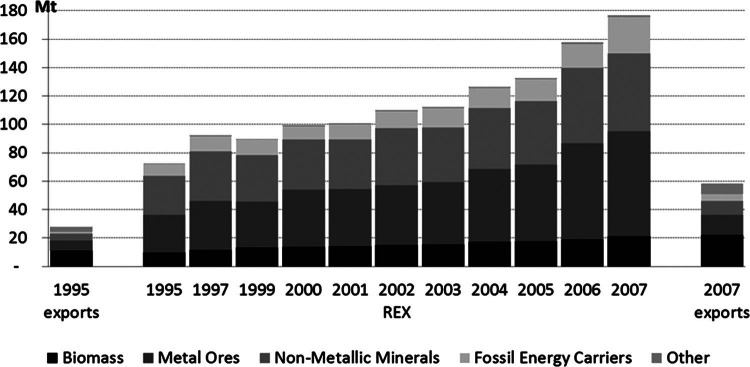
Austria's exports and RME of exports (REX) in million tonnes (Mt).

Between 1995 and 2007, relative growth of Austria's exports and RME of exports (REX) was almost identical (factor of 2.1 and 2.4, respectively). In 1995, REX amounted to approximately 73 million tonnes and increased to 177 million tonnes by 2007 (figure 5). Metals, nonmetallic minerals, and fossil energy carriers are the typical inputs into industrial production processes. For these material categories, REX was between 5.2 and 5.7 times larger than the exports. The ratio of REX to exports increased slightly during the observed period: Material corresponding to 2.6 (1995) and 3.0 (2007) times the mass of the exported goods themselves was used in the Austrian economy in their production.

As shown in figure 2, the importance of trade grew noticeably between 1995 and 2007. This is true not only for the contribution of imports to meeting domestic demand, but also to producing exports. Across the 12‐year period, exports grew by a factor of 2. The strongest growth occurred for the smallest material category in REX: fossil energy carriers (factor of 4.9 for exports and factor of 3.0 in REX). This growth may seem surprising at first, considering that Austria has very few domestic sources of fossil energy carriers and is dependent on imports to meet its demand. Petroleum refinery is, however, an important branch within the Austrian economy: It contributes a significant share to GDP and the oil and gas joint stock company, OMV, is the biggest Austrian company both in terms of sales and workforce. The refinery at Schwechat processes approximately 90% imported and 10% domestic petroleum resources; roughly 20% of the production is exported (Fachverband der Mineralölindustrie Österreichs 2010).

**Figure 6:**
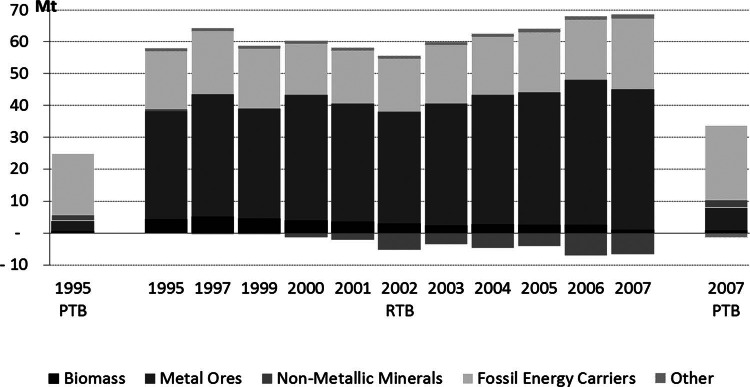
Austria's raw material and physical trade balance (RTB and PTB) in million tonnes (Mt).

Austria's physical trade balance (PTB = imports – exports) is positive, meaning that the economy is a net importer and global consumer of material. In 2007, Austria's PTB was slightly above 32 million tonnes, to which fossil energy carriers contributed the major share (23 million tonnes or 73%). The second‐largest material group in the PTB was metal ores (22%). Fossil energy carriers and metals are resources with limited domestic availability in Austria. For all other material categories, the PTB is comparatively small. For biomass, both import (23 million tonnes) and export (22 million tonnes) flows are considerable, but almost balanced, resulting in a PTB of just 0.9 million tonnes (see figure 6).

In 2007, Austria's raw material trade balance (RTB = RIM – REX) was about double the PTB (factor of 1.9). The large amount of metal ores required as inputs for the production of imported goods accounts for the major part of this difference. RIM exceeded REX of metals by 50 million tonnes. The next‐largest fraction in the RTB is fossil energy carriers (22 million tonnes). Between 1995 and 2007, Austria went from being a net importer of nonmetallic minerals RME to a net exporter: Whereas the PTB of nonmetallic minerals was 2 million tonnes, the RTB was –6.6 million tonnes in 2007. In both PTB and RTB, biomass is a category of (very small) net imports (0.8 and 1.1 million tonnes for PTB and RTB). From 1995 to 2007, the PTB grew from approximately 25 to 32 million tonnes by a factor of 1.3, whereas the RTB grew slightly less pronouncedly from approximately 58 to 62 million tonnes (factor of 1.1). This is because of the fact that Austria's exports and REX grew more pronouncedly than imports and RIM.

### Austria's Material Consumption

**Figure 7:**
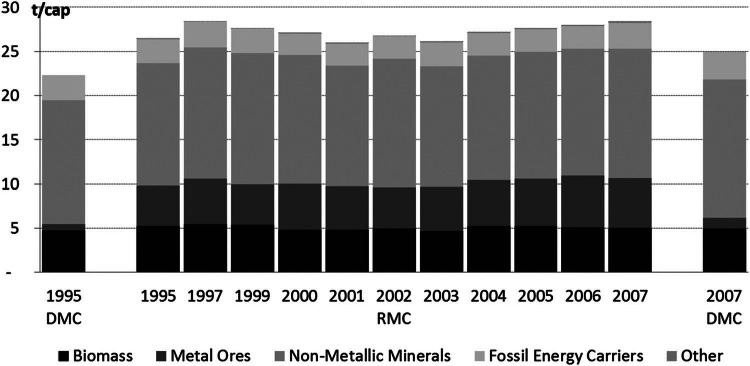
Austria's domestic and raw material consumption (DMC and RMC) between 1995 and 2007 in tonnes per capita (t/cap).

Between 1995 and 2007, Austrian DMC (DMC = DE + imports – exports) grew from 177 to 206 million tonnes by a factor of 1.2. This corresponds to a slight growth from 22 to 25 tonnes per capita and year (t/cap/a; figure 7). The major share of DMC consisted of nonmetallic minerals (16 t/cap/a, 63% of DMC), followed by biomass (5 t/cap/a, 20% of DMC) and fossil energy carriers (3 t/cap/a, 13% of DMC). Calculating DMC in terms of its RME renders the indicator raw material consumption (RMC = DE + RIM – REX), quantifying all raw materials used to satisfy Austrian final demand. In 2007, RMC reached a total of approximately 236 million tonnes, corresponding to 28 t/cap/a. RMC was 1.1 times or 4 t/cap/a higher than DMC. Nonmetallic minerals made up the major share (52% of RMC) and were followed by metal ores (20%), biomass (18%), and fossil energy carriers (10%).

Austria indirectly uses additional resources in other economies to satisfy domestic final demand. Though this outsourcing of material requirements protects the domestic environment, it contributes to higher environmental impacts at the global scale. For example, Austria's imports of fossil energy carriers in their RME amounted to approximately 48 million tonnes in 2007, which is 19 million tonnes more than the weight of imports crossing the border. If we convert this into carbon dioxide (CO_2_) emissions, we find that it corresponds to an additional 174 million tonnes (and a total of 433 million tonnes) of CO_2_^12^ that are emitted in other economies as a result of exports to Austria.

## Comparison of Results

In calculating the RME of Austrian trade, we developed the hybrid from a SRIO approach. In the latter, we applied the IO structure of the Austrian economy to imported goods (see above).

**Figure 8:**
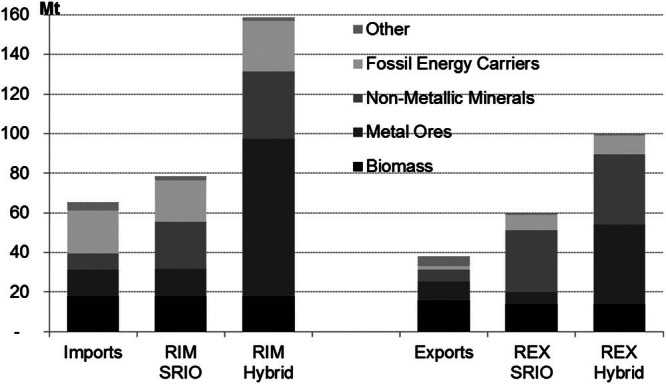
Comparison of the results of a single‐region input‐output (SRIO) and a hybrid approach to the calculation of Austria's raw material equivalents of imports (RIM) and of exports (REX) in million tonnes (Mt), 2000.

Figure 8 illustrates that if Austria had substituted all of its competitive and noncompetitive imported goods with domestic production under the given IO structures in 2000 (SRIO approach), less material input would have been required than by the import of these goods from other economies (hybrid approach). This, of course, presupposes that Austria is able to extract and produce all of its imported goods domestically under the prevailing production structure and must therefore be considered a hypothetical approximation. Imports amounted to approximately 65 million tonnes. Using the SRIO approach, the RIM were calculated to be 78 million tonnes (i.e. higher by a factor of 1.2). The RIM using a hybrid approach amount to approximately 159 million tonnes and were 2.4 times higher than the imports and 2.0 times higher than the SRIO RIM. Because some of these imported goods are, in turn, used for the production of exported goods and RIM is used in the calculation of REX, the SRIO approach also yields lower results for REX. In 2000, 38 million tonnes of material were exported. This corresponded to SRIO REX of 60 million tonnes and hybrid REX of 100 million tonnes. By using a hybrid approach, we were able to better account for the international production of Austria's noncompetitive imports, especially in those cases in which the weight of the import itself differs greatly from the weight of the extracted resource (e.g., metal concentrates imported as opposed to gross ore extracted).

Two other studies exist that also use a hybrid approach in calculating RME: one for the Czech Republic (Weinzettel and Kovanda 2009) and one for Germany (Buyny 2009; Buyny et al. 2009). The latter study also uses the relation between DMI and total supply in determining F, whereas the former relates DE to domestic supply. A comparison of results for 2003, when data are available for all three case studies, reveals that the difference between DMC and RMC is similar for the three industrialized central European economies: RMC exceeds DMC by a factor of 1.1 to 1.4. Overall, Germany and the Czech Republic exhibit a similar level of per‐capita material use (RMC in Germany: 23 t/cap/a; Czech Republic: 22 t/cap/a), whereas Austria has a higher material consumption (RMC, 29 t/cap/a). This is especially the result of a considerably higher level of consumption of nonmetallic minerals in Austria. This is, at least partly, an effect of data quality and estimation procedures: The Austrian statistical office recently implemented a new estimation method for construction mineral consumption, which significantly reduces those underestimations of extraction common for most countries.^13^ This improved data coverage and increased total material use (DMC) of Austria by 4 tonnes or a factor of 1.2 to 23 t/cap/a in 2003.

## Conclusions

Austria is a small, open economy that heavily relies on international trade. Exports contributed 59% to GDP in 2007 and growth in exports surpassed GDP growth by a factor of 1.6 between 1980 and 2010 (Statistik Austria 2012). At the same time, 34% of all materials used in Austria were imported in 2008 (Statistik Austria 2011b). Trade plays an increasingly important role in meeting the economy's resource demand and in maintaining its economic dynamic. One of the main issues that drove us to examine the raw material equivalents of trade was the question of whether productivity gains in industrialized economies may, in fact, be the result of outsourcing of material consumption (Weisz et al. 2008). We have been able to show that Austria's RMC is higher than its DMC (30 million tonnes or a factor of 1.1 in 2007), that is, that a share of the material consumption related to Austria's final demand occurs in other economies. We have also found that the dynamics of increasing dependence on resource imports from other economies do not translate into an equally dynamic growth of RMC. Instead, the latter figure stagnates across the period of investigation, exhibiting similar behavior as the DMC. This means that the trend in Austria's resource productivity measured using the RMC would be very similar to the trend based on the DMC. Austria is a net importer of biomass, metals, fossil energy carriers, and nonmetallic minerals, and the production of these imported goods requires material inputs in other economies. At the same time, imported raw materials are used in the production of exports, so that whereas outsourcing of production steps does occur, not all of it is related to Austrian final demand. Our results clearly show that resource use indicators, which do not take upstream requirements into account, may suffer from significant distortion. Strategies for sustainable resource use, which set targets for improvements in material productivity or reductions in material use such as those currently developed in the European Union, need to take the impact of production for trade into account. To this end, robust indicators and data are required. Only then will it be possible to monitor whether gains in resource productivity or reductions in material consumption could be the result of leakage effects (cf. Peters et al. 2011 for CO_2_). The pattern of outsourcing is not simply bilateral, but forms an intricate global web that becomes increasingly complex with the deeper integration of all economies into the global market. RME data and indicators are also relevant in addressing environmentally unequal exchange and the outsourcing of environmental burdens in the context of policies on sustainable trade relations.

In order for RME indicators to contribute to a better understanding of trade relations, a method for their calculation must be found that is internationally implementable and can be incorporated into the annual reporting of the national statistical institutes. In this article, we have presented a hybrid approach that, on the one hand, significantly improves the reliability of RME calculations, as compared to SRIO approaches. On the other hand, this approach still has its shortcomings, especially by assuming domestic production technology for competitive imports, which probably leads to a considerable distortion. Our calculations have provided us with a first better insight into Austria's share in global material use, simultaneously highlighting the necessity of improving methods of RME calculations. The consideration of the RME of an economy's trade are a prerequisite to evaluating both its environmental impact and its resource efficiency.

## Supplementary Information

**Supporting Information S1:** This supporting information contains 6 tables. Table S1 defines the abbreviations used in the supporting information. Table S2 provides data tables from 1995–2007. Table S3 shows ratios of RME flow to non‐RME flows. Table S4 lists sectors of economic activities. Tables S5 and S6 show the allocation of domestic extraction and imports, respectively, to activities in 2007.

Supporting info item

## Footnotes

1 For a review of the development of the MRIO approach, see Wiedmann (2009).

2 Full coverage is hindered by truncation decisions, which have to be made in the tracing of any life cycle. Allocation decisions can potentially lead to double counting, but must be made whenever one production process produces more than one good. Allocation can be based on different aspects of the products (economic value, mass/energy, or material input avoided by coupled production. The results of these different allocation approaches can be completely contrary, so that the decision as to which procedure is used has a major impact on the results (Chapman 1974; Wilting 1996).

3 Materials displaced during extraction activities, but not extracted themselves (e.g., overburden moved during mining activities). Unused extraction is not subject to further processing.

4 The term “competitive imports” refers to imported goods, which are similar to (and therefore in competition to) goods produced domestically.

5 For the calculation of these coefficients, UN Comtrade data (and not the MFA data provided by Statistics Austria) was used because it offered the necessary amount of detail to characterize Austrian imports by product group. In case of discrepancies between the two data sources, the UN Comtrade data was extrapolated to match the sums reported by Statistics Austria.

6 The term “waste rock” is used here to refer to the run of mine (i.e., to the surrounding rock in the metal ore), which is extracted from the mine and removed during further processing steps. It does not include the overburden, which may be moved but is not extracted, during mining.

7 Allocation tables are provided in the Supporting Information on the Web.

8 The category “other products” had to be introduced because some of the product groups that exist in the IO data are composed of more than one type of the four basic materials, and a main fraction cannot be unambiguously identified.

9 One tonne (t) = 10^3^ kilograms (kg, SI) ≈ 1.102 short tons.

10 Following MFA conventions, we accounted for metals domestic extraction as the gross ore and not as metal content (see Eurostat 2001).

11 Please also refer to the Supporting Information on the Web for an overview of the ratios between RIM and imports.

12 For the estimation, we used an average conversion factor of 9.1 tonnes of CO_2_ equivalent per tonne of FF use (based on the Austrian import‐mix) (Öko‐Institut 2009).

13 Previously, significant amounts of resource extraction by construction activities were previously not reported by standard statistics. Underestimations were the result of (1) confidential data, (2) reporting procedures (i.e., enterprises below 20 employees have no reporting obligation), and (3) noncharacteristic production in the construction activities, which is not reported as domestic extraction and thus was not included in material flow accounts (Milota et al. 2011).
