# Supplementary material for: Consumption‐based Material Flow Accounting: Austrian Trade and Consumption in Raw Material Equivalents 1995–2007
Source: J Ind Ecol. 2013 Sep 30;18(1):102–12. doi: 10.1111/jiec.12055 (PMC13056745; doi:10.1111/jiec.12055)
Supplement: Supplementary file 1 — Supporting info item [file 44498_2014_1801015_MOESM1_ESM.pdf]

SUPPORTING INFORMATION FOR:

Schaffartzik, A., N. Eisenmenger, F. Krausmann, and H. Weisz. 2013. Consumption-based material flow accounting: Austrian trade and consumption in raw material equivalents 1995-2007. *Journal of Industrial Ecology*.

Summary

This supporting information contains 6 tables. Table S1 defines the abbreviations used in the supporting information. Table S2 provides data tables from 1995-2007. Table S3 shows ratios of RME flow to non-RME flows. Table S4 lists sectors of economic activities. Tables S5 and S6 show the allocation of domestic extraction and imports, respectively, to activities in 2007.

Contents

Table S1: Abbreviations Used in the Supporting Information 2

Table S2: Data Tables 1995-2007 3

Table S3: Ratios of RME Flows to Non-RME Flows 4

Table S4: Sectors of Economic Activities (ÖNACE 2002) 5

Table S5: Allocation of Domestic Extraction to Activities 2007 6

Table S6: Allocation of Imports to Activities 2007 7

Table S1: Abbreviations Used in the Supporting Information

Abbreviations		
	Definition	Calculation (if applicable)
**DE**	domestic extraction	
**DMC**	domestic material consumption	DMC = DE + PTB
**DMI**	direct material input	DMI = DE + imports
**GDP**	gross domestic product	
**MFA**	material flow accounting	
**pop**	population	
**PTB**	physical trade balance	PTB = imports - exports
**REX**	raw material equivalents of exports	
**RIM**	raw material equivalents of imports	
**RMC**	raw material consumption	RMC = DE + RTB
**RME**	raw material equivalents	
**RMI**	raw material input	RMI = DE + RIM
**RTB**	raw material trade balance	RTB = RIM - REX

Table S2: Data Tables 1995-2007

Data Tables	1995	2000	2005	2007
**Supplementary Data (Source: Statistik Austria 2012)**				
pop [1000]	7 948	8 012	8 225	8 301
GDP [mio Euro]	194 301	225 097	243 585	260 952
**MFA Data (Source: Statistik Austria 2011)**				
**DE [kt]**	**152 787**	**158 639**	**167 285**	**173 753**
**Imports [kt]**	**52 529**	**65 364**	**81 295**	**90 539**
**RIM [kt]**	**130 422**	**158 513**	**192 908**	**238 925**
**Exports [kt]**	**28 006**	**37 841**	**49 845**	**58 438**
**REX [kt]**	**72 634**	**99 652**	**132 882**	**176 842**
**PTB [kt]**	**24 523**	**27 523**	**31 450**	**32 101**
**RTB [kt]**	**57 789**	**58 861**	**60 026**	**62 083**
**DMC [kt]**	**177 310**	**186 162**	**198 735**	**205 854**
Biomass	37 966	36 526	40 701	41 417
Metal Ores	5 492	6 359	7 653	9 611
Non-Metallic Minerals	111 269	120 021	124 316	130 368
Fossil Energy Carriers	22 748	23 948	27 397	25 809
Other Products	- 165	- 692	- 1 332	- 1 351
**RMC [kt]**	**210 576**	**217 500**	**227 311**	**235 836**
Biomass	41 799	38 522	43 028	41 645
Metal Ores	36 081	41 580	43 990	46 499
Non-Metallic Minerals	110 078	116 699	118 038	121 635
Fossil Energy Carriers	21 713	19 656	20 980	24 658
Other Products	904	1 043	1 275	1 399
**DMI [kt]**	**205 316**	**224 003**	**248 580**	**264 292**
Biomass	49 239	52 263	61 048	63 545
Metal Ores	12 371	15 831	19 720	23 864
Non-Metallic Minerals	116 374	126 174	131 761	139 884
Fossil Energy Carriers	23 774	25 450	30 720	30 886
Other Products	3 558	4 285	5 331	6 113
**RMI [kt]**	**283 209**	**317 152**	**360 193**	**412 678**
Biomass	51 638	52 221	60 826	63 111
Metal Ores	62 766	81 864	97 706	120 334
Non-Metallic Minerals	137 160	152 151	162 806	176 343
Fossil Energy Carriers	30 160	29 048	36 388	50 030
Other Products	1 486	1 867	2 467	2 861

Table S3: Ratios of RME Flows to Non-RME Flows

	Ratios			
Unit: kt/kt	1995	2000	2005	2007
**RIM/Imports**	**2.5**	**2.4**	**2.4**	**2.6**
**REX/Exports**	**2.6**	**2.6**	**2.7**	**3.0**
**RMC/DMC**	**1.2**	**1.2**	**1.1**	**1.1**
**Biomass**	1.1	1.1	1.1	1.0
**Metal Ores**	6.6	6.5	5.7	4.8
**Non-Metallic Minerals**	1.0	1.0	0.9	0.9
**Fossil Energy Carriers**	1.0	0.8	0.8	1.0
**Other**	- 5.5	- 1.5	- 1.0	- 1.0

Table S4: Sectors of Economic Activities (ÖNACE 2002)

	Economic Activites
Code	Element
01	Crop and animal production, hunting and related service activities
02	Forestry and logging
05	Fishing and aquaculture
10	Mining of coal and lignite
11	Extraction of crude petroleum and natural gas
14	Mining of metal ores and other mining and quarrying
15	Manufacture of food products and beverages
16	Manufacture of beverages
17	Manufacture of tobacco products
18	Manufacture of textiles
19	Manufacture of wearing apparel
20	Manufacture of leather and related products
21	Manufacture of wood and of products of wood and cork, except
22	Manufacture of paper and paper products
23	Printing and reproduction of recorded media
24	Manufacture of coke and refined petroleum products
25	Manufacture of chemicals and chemical products
26	Manufacture of rubber and plastic products
27	Manufacture of other non-metallic mineral products
28	Manufacture of basic metals
29	Manufacture of fabricated metal products, except machinery and
30	Manufacture of machinery and equipment n.e.c.
31	Manufacture of office equipment
32	Manufacture of electrical equipment
33	Telecommunications
34	Manufacture of computer, electronic and optical products
35	Manufacture of motor vehicles, trailers and semi-trailers
36	Manufacture of other transport equipment
37	Manufacture of furniture
40	Materials recovery
41	Electricity, gas, steam and air conditioning supply
45	Water collection, treatment and supply
50	Construction of buildings, civil engineering
51	Wholesale and retail trade and repair of motor vehicles and
52	Wholesale trade, except of motor vehicles and motorcycles
55	Retail trade, except of motor vehicles and motorcycles
60	Accommodation, food and beverage service activities
61	Land transport and transport via pipelines
62	Water transport
63	Air transport
64	Warehousing and support activities for transportation
65	Postal and courier activities
66	Financial service activities, except insurance and pension funding
67	Insurance, reinsurance and pension funding, except compulsory
70	Activities auxiliary to financial services and insurance activities
71	Real estate activities
72	Rental and leasing activities
73	Computer programming, consultancy and related activities
74	Scientific research and development
75	Other professional, scientific and technical activities
80	Public administration and defence; compulsory social security
85	Education
90	Human health activities, residential care activities, social work
91	Waste disposal, sewage
92	Activities of membership organisations
93	Creative, arts and entertainment activities, libraries, archives,
95	Private households

Table S5: Allocation of Domestic Extraction to Activities 2007

Allocation of MFA Domestic Extraction to Economic Activities 2007									
		Sectors (CPA)							
MFA Code	MFA Name	1	2	5	10	11	13	14	45
1.1.1.	Cereals	1.0							
1.1.10.	Other crops	1.0							
1.1.2.	Roots, tubers	1.0							
1.1.3.	Sugar crops	1.0							
1.1.4.	Pulses	1.0							
1.1.5.	Nuts	1.0							
1.1.6.	Oil bearing crops	1.0							
1.1.7.	Vegetables	1.0							
1.1.8.	Fruits	1.0							
1.1.9.	Fibres	1.0							
1.2.1.	Crop residues (used)	1.0							
1.2.2.	Fodder crops and grazed biomass	1.0							
1.3.1.	Timber (industrial roundwood)		1.0						
1.3.2.	Wood fuel and other extraction		1.0						
1.4.1.	Fish catch			1.0					
1.5.	Hunting and gathering	1.0							
2.1.	Iron ores						1.0		
2.2.1.	Copper ores						1.0		
2.2.2.	Nickel ores						1.0		
2.2.3.	Lead ores						1.0		
2.2.4.	Zinc ores						1.0		
2.2.5.	Tin ores						1.0		
2.2.6.	Gold, silver, platinum, and other precious metal ores						1.0		
2.2.7.	Bauxite and other aluminum ores						1.0		
2.2.9.	Other metal ores						1.0		
3.1.1.	Ornamental or building stone							1.0	
3.1.2.	Chalk and dolomite							1.0	
3.1.3.	Slate							1.0	
3.1.4.	Chemical and fertilizer minerals							1.0	
3.1.5.	Salt							1.0	
3.1.6.	Other mining and quarrying products							1.0	
3.2.1.	Limestone and gypsum							0.5	0.5
3.2.2.	Gravel and sand							0.5	0.5
3.2.3.	Clays and kaolin							0.5	0.5
4.1.1.	Brown coal				1.0				
4.1.2.	Hard coal				1.0				
4.1.4.	Peat				1.0				
4.2.1.1.	Crude Oil					1.0			
4.2.2.	Natural gas					1.0			

Table S6: Allocation of Imports to Activities 2007

Allocation of MFA Imports to Economic Activities 2007 (Part 1)												
MFA Code	MFA Name	1	2	5	10	11	13	14	15	16	17	18
1.1.1.	Cereals	0.7	-	-	-	-	-	-	0.3	-	-	-
1.1.10.	Other crops	0.1	-	-	-	-	-	-	0.8	0.0	-	-
1.1.2.	Roots, tubers	-	-	-	-	-	-	-	1.0	-	-	-
1.1.3.	Sugar crops	-	-	-	-	-	-	-	1.0	-	-	-
1.1.4.	Pulses	0.7	-	-	-	-	-	-	0.3	-	-	-
1.1.5.	Nuts	0.6	-	-	-	-	-	-	0.4	-	-	-
1.1.6.	Oil bearing crops	1.0	-	-	-	-	-	-	-	-	-	-
1.1.7.	Vegetables	0.7	-	-	-	-	-	-	0.3	-	-	-
1.1.8.	Fruits	0.6	-	-	-	-	-	-	0.4	-	-	-
1.1.9.	Fibres	0.1	-	-	-	-	-	-	0.9	-	-	-
1.2.1.	Crop residues (used)	-	-	-	-	-	-	-	1.0	-	-	-
1.2.2.	Fodder crops and grazed biomass	0.7	-	-	-	-	-	-	0.3	-	-	-
1.3.1.	Timber (industrial roundwood)	-	-	-	-	-	-	-	-	-	-	-
1.3.2.	Wood fuel and other extraction	0.0	0.5	-	-	-	-	-	-	-	-	-
1.4.1.	Fish catch	-	-	0.4	-	-	-	-	0.6	-	-	-
1.6.1.	Live animals	1.0	-	-	-	-	-	-	-	-	-	-
1.6.2.	Meat and meat preparations	-	-	-	-	-	-	-	1.0	-	-	-
1.6.3.	Dairy products, birds' eggs, honey	-	-	-	-	-	-	-	1.0	-	-	-
1.6.4.	Other products from animals	0.0	-	-	-	-	-	-	0.9	-	-	0.0
1.7.	Products mainly from biomass	0.0	-	-	-	-	-	-	0.2	-	-	-
2.1.	Iron ores	-	-	-	-	-	0.6	-	-	-	-	-
2.2.1.	Copper ores	-	-	-	-	-	0.0	-	-	-	-	-
2.2.2.	Nickel ores	-	-	-	-	-	0.0	-	-	-	-	-
2.2.3.	Lead ores	-	-	-	-	-	-	-	-	-	-	-
2.2.4.	Zinc ores	-	-	-	-	-	-	-	-	-	-	-
2.2.5.	Tin ores	-	-	-	-	-	-	-	-	-	-	-
2.2.6.	Gold, silver, platinum, and other precious metal ores	-	-	-	-	-	-	-	-	-	-	-
2.2.7.	Bauxite and other aluminum ores	-	-	-	-	-	0.1	-	-	-	-	-
2.2.9.	Other metal ores	-	-	-	-	-	0.1	-	-	-	-	-
2.3.	Products mainly from metals	-	-	-	-	-	-	-	-	-	-	-
3.1.1.	Ornamental or building stone	-	-	-	-	-	-	1.0	-	-	-	-
3.1.2.	Chalk and dolomite	-	-	-	-	-	-	0.3	-	-	-	-
3.1.3.	Slate	-	-	-	-	-	-	1.0	-	-	-	-
3.1.4.	Chemical and fertilizer minerals	-	-	-	-	-	-	0.2	-	-	-	-
3.1.5.	Salt	-	-	-	-	-	-	1.0	-	-	-	-
3.1.6.	Other mining and quarrying products	-	-	0.0	-	-	-	0.2	-	-	-	-
3.2.2.	Gravel and sand	-	-	-	-	-	-	1.0	-	-	-	-
3.2.3.	Clays and kaolin	-	-	-	-	-	-	0.4	-	-	-	-
3.3.	Products mainly from non-metallic minerals	-	-	-	-	-	-	-	-	-	-	-
4.1.1.	Brown coal	-	-	-	1.0	0.0	-	-	-	-	-	-
4.1.2.	Hard coal	-	-	-	1.0	-	-	-	-	-	-	-
4.1.4.	Peat	-	-	-	1.0	0.0	-	-	-	-	-	-
4.2.1.1.	Crude Oil	-	-	-	-	0.5	-	-	-	-	-	-
4.2.2.	Natural gas	-	-	-	-	1.0	-	-	-	-	-	-
4.3.	Products mainly from petroleum products	-	-	-	-	-	-	-	-	-	-	-
5.	Other products	-	-	-	-	-	-	-	0.0	-	0.1	0.0

Allocation of MFA Imports to Economic Activities 2007 (Part 2)

MFA Code	MFA Name	19	20	21	23	24	25	26	27	28	29
1.1.1.	Cereals	-	-	-	-	-	-	-	-	-	-
1.1.10.	Other crops	-	-	-	-	-	-	-	-	-	-
1.1.2.	Roots, tubers	-	-	-	-	-	-	-	-	-	-
1.1.3.	Sugar crops	-	-	-	-	-	-	-	-	-	-
1.1.4.	Pulses	-	-	-	-	-	-	-	-	-	-
1.1.5.	Nuts	-	-	-	-	-	-	-	-	-	-
1.1.6.	Oil bearing crops	-	-	-	-	-	-	-	-	-	-
1.1.7.	Vegetables	-	-	-	-	-	-	-	-	-	-
1.1.8.	Fruits	-	-	-	-	-	-	-	-	-	-
1.1.9.	Fibres	-	-	-	-	-	-	-	-	-	-
1.2.1.	Crop residues (used)	-	-	-	-	-	-	-	-	-	-
1.2.2.	Fodder crops and grazed biomass	-	-	-	-	-	-	-	-	-	-
1.3.1.	Timber (industrial roundwood)	-	1.0	-	-	-	-	-	-	-	-
1.3.2.	Wood fuel and other extraction	-	0.3	-	-	-	0.2	-	-	-	-
1.4.1.	Fish catch	-	-	-	-	-	-	-	-	-	-
1.6.1.	Live animals	-	-	-	-	-	-	-	-	-	-
1.6.2.	Meat and meat preparations	-	-	-	-	-	-	-	-	-	-
1.6.3.	Dairy products, birds' eggs, honey	-	-	-	-	-	-	-	-	-	-
1.6.4.	Other products from animals	0.1	-	-	-	-	-	-	-	-	-
1.7.	Products mainly from biomass	-	-	0.8	-	-	-	-	-	-	-
2.1.	Iron ores	-	-	-	-	-	-	-	0.4	-	-
2.2.1.	Copper ores	-	-	-	-	-	-	-	1.0	-	-
2.2.2.	Nickel ores	-	-	-	-	-	-	-	1.0	-	-
2.2.3.	Lead ores	-	-	-	-	-	-	-	1.0	-	-
2.2.4.	Zinc ores	-	-	-	-	-	-	-	1.0	-	-
2.2.5.	Tin ores	-	-	-	-	-	-	-	1.0	-	-
2.2.6.	Gold, silver, platinum, and other precious metal ores	-	-	-	-	-	-	-	1.0	-	-
2.2.7.	Bauxite and other aluminum ores	-	-	-	-	-	-	-	0.9	-	-
2.2.9.	Other metal ores	-	-	-	-	-	-	-	0.9	-	-
2.3.	Products mainly from metals	-	-	-	-	-	-	-	0.0	0.3	0.3
3.1.1.	Ornamental or building stone	-	-	-	-	-	-	-	-	-	-
3.1.2.	Chalk and dolomite	-	-	-	-	-	-	0.7	-	-	-
3.1.3.	Slate	-	-	-	-	-	-	-	-	-	-
3.1.4.	Chemical and fertilizer minerals	-	-	-	-	0.8	-	-	-	-	-
3.1.5.	Salt	-	-	-	-	-	-	-	-	-	-
3.1.6.	Other mining and quarrying products	-	-	-	0.0	0.5	-	0.2	0.1	-	-
3.2.2.	Gravel and sand	-	-	-	-	-	-	-	-	-	-
3.2.3.	Clays and kaolin	-	-	-	-	-	-	0.6	-	-	-
3.3.	Products mainly from non-metallic minerals	-	-	-	-	-	-	1.0	-	-	-
4.1.1.	Brown coal	-	-	-	-	-	-	-	-	-	-
4.1.2.	Hard coal	-	-	-	-	-	-	-	-	-	-
4.1.4.	Peat	-	-	-	-	-	-	-	-	-	-
4.2.1.1.	Crude Oil	-	-	-	0.5	-	-	-	-	-	-
4.2.2.	Natural gas	-	-	-	0.0	-	-	-	-	-	-
4.3.	Products mainly from petroleum products	-	-	-	0.3	0.6	0.1	-	-	-	-
5.	Other products	0.0	0.0	0.0	-	0.4	0.1	0.0	-	-	0.0

Allocation of MFA Imports to Economic Activities 2007 (Part 3)

MFA Code	MFA Name	30	31	32	33	34	35	36	40	92	93
1.1.1.	Cereals	-	-	-	-	-	-	-	-	-	-
1.1.10.	Other crops	-	-	-	-	-	-	-	-	-	-
1.1.2.	Roots, tubers	-	-	-	-	-	-	-	-	-	-
1.1.3.	Sugar crops	-	-	-	-	-	-	-	-	-	-
1.1.4.	Pulses	-	-	-	-	-	-	-	-	-	-
1.1.5.	Nuts	-	-	-	-	-	-	-	-	-	-
1.1.6.	Oil bearing crops	-	-	-	-	-	-	-	-	-	-
1.1.7.	Vegetables	-	-	-	-	-	-	-	-	-	-
1.1.8.	Fruits	-	-	-	-	-	-	-	-	-	-
1.1.9.	Fibres	-	-	-	-	-	-	-	-	-	-
1.2.1.	Crop residues (used)	-	-	-	-	-	-	-	-	-	-
1.2.2.	Fodder crops and grazed biomass	-	-	-	-	-	-	-	-	-	-
1.3.1.	Timber (industrial roundwood)	-	-	-	-	-	-	-	-	-	-
1.3.2.	Wood fuel and other extraction	-	-	-	-	-	-	-	-	-	-
1.4.1.	Fish catch	-	-	-	-	-	-	-	-	-	-
1.6.1.	Live animals	-	-	-	-	-	-	-	-	-	-
1.6.2.	Meat and meat preparations	-	-	-	-	-	-	-	-	-	-
1.6.3.	Dairy products, birds' eggs, honey	-	-	-	-	-	-	-	-	-	-
1.6.4.	Other products from animals	-	-	-	-	-	-	-	-	-	-
1.7.	Products mainly from biomass	-	-	-	-	-	-	-	-	-	0.0
2.1.	Iron ores	-	-	-	-	-	-	-	-	-	-
2.2.1.	Copper ores	-	-	-	-	-	-	-	-	-	-
2.2.2.	Nickel ores	-	-	-	-	-	-	-	-	-	-
2.2.3.	Lead ores	-	-	-	-	-	-	-	-	-	-
2.2.4.	Zinc ores	-	-	-	-	-	-	-	-	-	-
2.2.5.	Tin ores	-	-	-	-	-	-	-	-	-	-
2.2.6.	Gold, silver, platinum, and other precious metal ores	-	-	-	-	-	-	-	-	-	-
2.2.7.	Bauxite and other aluminum ores	-	-	-	-	-	-	-	-	-	-
2.2.9.	Other metal ores	-	-	-	-	-	-	-	-	-	-
2.3.	Products mainly from metals	0.0	0.1	0.0	0.0	0.3	0.1	0.0	-	-	-
3.1.1.	Ornamental or building stone	-	-	-	-	-	-	-	-	-	-
3.1.2.	Chalk and dolomite	-	-	-	-	-	-	-	-	-	-
3.1.3.	Slate	-	-	-	-	-	-	-	-	-	-
3.1.4.	Chemical and fertilizer minerals	-	-	-	-	-	-	-	-	-	-
3.1.5.	Salt	-	-	-	-	-	-	-	-	-	-
3.1.6.	Other mining and quarrying products	-	-	-	-	-	-	-	-	-	-
3.2.2.	Gravel and sand	-	-	-	-	-	-	-	-	-	-
3.2.3.	Clays and kaolin	-	-	-	-	-	-	-	-	-	-
3.3.	Products mainly from non-metallic minerals	-	-	-	-	-	-	-	-	-	-
4.1.1.	Brown coal	-	-	-	-	-	-	-	-	-	-
4.1.2.	Hard coal	-	-	-	-	-	-	-	-	-	-
4.1.4.	Peat	-	-	-	-	-	-	-	-	-	-
4.2.1.1.	Crude Oil	-	-	-	-	-	-	-	-	-	-
4.2.2.	Natural gas	-	-	-	-	-	-	-	-	-	-
4.3.	Products mainly from petroleum products	-	-	-	-	-	-	-	-	-	-
5.	Other products	-	0.0	-	0.0	-	-	0.2	-	0.0	-

References

Statistik Austria. 2011. Materialflussrechnung. http://www.statistik.at/web_de/statistiken/energie_und_umwelt/umwelt/materialflussrechnung/index.html. Accessed 12/2012.

Statistik Austria. 2012. STATcube Statistical Database. http://www.statistik.at/web_de/services/datenbank_superstar/aufruf/index.html. Accessed 12/2012.
